# Effectiveness of the refined health literacy course on improving the health literacy competencies of undergraduate nursing students: quantitative and qualitative perspectives

**DOI:** 10.1080/10872981.2023.2173042

**Published:** 2023-01-30

**Authors:** Mei-Chuan Chang, Jui-Hung Yu, Jyh-Gang Hsieh, Mi-Hsiu Wei, Ying-Wei Wang

**Affiliations:** a Department of Nursing, College of Medicine, Tzu Chi University, Hualien City, Taiwan; b Department of Public Health, Tzu Chi University, Hualien City, Taiwan; c Department of Family Medicine, Hualien Tzu Chi Hospital, Hualien City, Taiwan; d Department of Medical Humanities, School of Medicine, Tzu Chi University, Hualien City, Taiwan; e Department of Communication Studies, Tzu Chi University, Hualien City, Taiwan

**Keywords:** Health literacy, competencies, course, undergraduate nursing students, team-based learning, effectiveness

## Abstract

People with limited health literacy comprise a high-risk group for adverse health outcomes. Nurses must be made aware of the importance of health literacy and communicate with patients in plain language, which will solve the obstacles for patients using health care services. Providing health literacy education for nurses is an important strategy for health literacy practices. This study aimed to develop a refined health literacy course for nursing students and evaluate its effectiveness. The study used a single-group pre- and post-test quasi-experimental design and conducted focus group interviews. The intervention was an eight-hour course and applied team-based learning combined with various teaching methods. The study enrolled second-year nursing students of a university in eastern Taiwan via convenient sampling. A self-reported structured questionnaire was used to compare the participants’ familiarity with health literacy, attitude, confidence in oral communication, and ability in written communication before and after the course. To discuss the learning experience of the course, the study held two focus groups with 12 participants. A total of 81 participants completed the pre- and post-test questionnaires. The results showed that familiarity with health literacy (*t*=9.12, *t*<.001), attitude (*t*=4.89, *t*<.001), confidence in oral communication (*t*=4.12, *t*<.001), and ability in written communication (*t*=8.83, *t*<.001) showed improvement after the course. Data analysis of focus group interviews yielded two categories and seven themes for the learning experience, most of which showed participants’ positive learning experiences in the health literacy course. The course effectively enhanced the nursing students’ knowledge and attitude toward health literacy and the ability to communicate with patients using health literacy principles. The results can provide a reference for integrating health literacy education into the undergraduate nursing curriculum.

## Background

Health literacy is a critical determinant of an individual’s health status [1] and a crucial issue for global health promotion [2]. United States Healthy People 2030 defines personal health literacy as ‘the degree to which individuals have the ability to find, understand, and use information and services to inform health-related decisions and actions for themselves and others’ [3]. Individuals with limited health literacy may face multiple challenges owing to difficulty understanding and applying health information. They may not understand their doctors’ explanations for illnesses, read drug labels and health education leaflets correctly, or know how to ask questions. All these problems could affect self-care and medical decision-making [4,5]. Regarding the issue of health literacy, the difficulty and complexity of messages in the healthcare environment in which individuals live are relevant factors [6]. To help patients with limited health literacy, healthcare professionals must communicate in plain language, confirm patients’ understanding of information, provide easy-to-read printed messages, and assist patients in medical decision-making. Therefore, developing healthcare professionals’ capabilities to follow health literacy practices is an important strategy to promote health-literate healthcare [7].

Developing such professionals requires a two-pronged approach: training healthcare workers at both on-the-job and pre-license stages [8,9]. While communication skills are a core part of the curriculum for educating health professional students, awareness of health literacy is not explicitly required [10]. In recent years, health literacy competencies for healthcare professionals have been proposed and defined [11–13], providing the basis for the design of health literacy education at various stages. Many studies on health literacy curricula for undergraduate health professional students have been published successively [14,15]. However, no clear consensus has been reached on the teaching content and how to teach it [16]. Indeed, health literacy education is not widely available to undergraduate health professional students. This is possible because of an overloaded curriculum that leaves little time for health literacy courses and a lack of research to reach a consensus on course content, course structure, and effective teaching methods [17]. Communication and health education skills are core competencies of nursing students as potential nursing workers. Incorporating health literacy in nursing courses can improve nursing students’ ability to care for those with limited health literacy, thus producing better health outcomes [18,19]. Therefore, exploring the feasibility of health literacy courses for nursing students and offering adequate evidence have practical implications for promoting health literacy courses.

Identifying health literacy competencies is the first important step in developing a health literacy course. Coleman, Hudson, and Maine defined health literacy educational competencies as ‘the knowledge, skills, and attitudes that health professionals need to effectively address low health literacy among consumers of health care services and health information.’ They proposed 62 health literacy competencies and 32 health literacy practices and suggested that these items must be prioritized for different medical fields [11]. Toronto conducted an e-Delphi study that identified 50 items of health literacy competencies for registered nurses. As for knowledge, experts assumed it crucial for nursing staff to know that regardless of health literacy level, clear communication and plain language are beneficial to everyone. In the skills domain, registered nurses should speak to patients slowly and clearly. In the attitude domain, registered nurses should exhibit the attitude that everyone has the risk of communication errors; thus, a universal precautions approach is warranted [12]. Coleman et al. proposed 32 health literacy practices of healthcare professionals and classified them into three groups by priority level. They suggested that the eight practices of the first group, such as avoiding medical jargon, applying ‘teach-back,’ and ‘patient-centered’ communication, should be listed as techniques routinely used by healthcare professionals to improve the clarity of communication between doctors and patients and alleviate the adverse effects of health literacy issues [20]. Hernes and Ott suggested that these eight items can be included in the health literacy education of undergraduate nursing students and can inform curriculum planning at the beginning of promoting health literacy education [16].

Meanwhile, questions remain on the teaching method and course content for existing health literacy courses. Studies show that most health professional schools include health literacy in their required curriculum and as a section of a specific course [14,21]. The course time can be as little as 20–50 minutes and as many as 45 hours [14,22]. The course content includes an introduction to the concept of health literacy, the prevalence of low health literacy, the correlation between health literacy and health outcomes, and various skills in oral communication, especially the use of plain language and teach-back approaches, patient-centered communication, which are covered in most cases. Only a few studies addressed the evaluation and development of written health education materials [14,21–23].

These studies also noted that multiple approaches are often used to teach health literacy content. Generally, didactic is supplemented by other activities, such as case discussion, role play, standardized patients, videos [14,21,22], and game-based learning [24]. Some contents also require after-school assignments to help students better understand what they have been taught and enhance health literacy skills [14,21,22].

Team-based learning (TBL) is an innovative teaching strategy emphasizing a learner-centered approach to learning. It has systematic and standard procedures, including pre-class preparation, readiness test, teacher feedback and clarification, and curriculum application. It enhances learning outcomes through the participation of team members [25]. With pre-class preparation and knowledge memorized through self-study, students can, in class time, conduct activities that emphasize the application of knowledge and enhance critical thinking instead of restating knowledge [26,27]. Studies have confirmed that compared with face-to-face teaching methods, TBL can improve students’ skills and abilities in communication, critical thinking, and problem-solving [28,29]. This teaching strategy has been widely used in nursing-related subjects [25].

With health literacy becoming a significant global health issue, it has been extensively studied in Taiwan. However, the topic of health literacy education for healthcare professionals has not been duly addressed, and studies on undergraduate health literacy education are exceptionally scarce. Regarding health literacy education, the present problems identified in the literature are the lack of a consensus on the scope of undergraduate health literacy courses and the excessive curriculum load. Thus, we refined the course to be essential in the content of knowledge and skills for health literacy with limited classroom time. The aims of this study were to evaluate the health literacy course’s effectiveness quantitatively and verify the quantitative results from qualitative perspectives. The research results can generalize the health literacy course in existing undergraduate nursing education.

## Methods

This study used a single-group pre- and post-test design to evaluate students’ learning outcomes before and after the course and then employed focus group interviews, which were analyzed to determine students’ self-described learning experiences. Both quantitative and qualitative data were collected to interpret the research results better.

### Participants

We used convenient sampling to enroll 113 nursing students who took the pediatric nursing course from a two-year nursing program at a university in eastern Taiwan. Pediatric nursing is a required course taught by researchers. We designed the health literacy content in the course. Eighty-one (71.7%) completed the course and filled out the pre- and post-test questionnaires. Two focus groups of 12 students were recruited from those who completed the course and questionnaires, with each group comprising six students.

### Intervention

The intervention of this study was a refined health literacy course that adopted TBL method with various teaching methods such as reality video simulation learning, health educational materials assessing exercise, and homework to complete course content with minimized teaching hours. The eight-hour health literacy content was incorporated into the pediatric nursing course and comprised of four-hour class activities and a four-hour oral presentation of students’ after-school assignments. Learning objectives and contents were developed by researchers according to Anderson et al.’s revision of Bloom’s taxonomy of educational objects in the cognitive process dimension, which has six objectives [30]. Table 1 presents the learning objectives derived from the aforementioned, as well as the teaching contents and learning activities decided by drawing from relevant literature. The researchers have rich experience in TBL teaching and health literacy research. The course content was reviewed and revised by three health literacy experts: a professor of health education and communication, an associate professor of nursing, and a senior family medicine physician.

**Table 1.** Learning objectives and contents of the intervention course.

Learning objectives	Course contents
Remember Know the definition of health literacy, prevalence of low health literacy, health outcomes, and promotion strategies	Unit 1: Health literacy overview, including the definition of health literacy, the prevalence of low health literacy and health outcomes, and the universal precautions approach
Understand Understand the principles of oral and written communication addressing health literacy	Unit 2: Oral communication, including relevant health literacy principles, teaching patients to ask questions, practices and applications of teach-back Unit 3: Written communication, including the introduction of evaluation indicators and guidelines for health education materials and the steps to develop health literacy appropriate education materials
Apply Apply knowledge in health literacy communication to clinical situations	Classroom activities Review a video of physician-patient oral communication in medical settings; criticize and discuss the communication problems in groups Evaluate the same health education leaflet in groups against the indicators for health education materials and discuss the evaluation results Homework Design oral communication scripts and develop written health education materials
Analyze Analyze problems in oral communication and written education materials according to the communication principles	Classroom activities (same as above)
Evaluate Evaluate problems in oral communication and written education materials according to the communication principles	Classroom activities (same as above)
Create Consolidate health literacy knowledge; design proper health literacy oral communication and written education materials	Homework (same as above)

After the content was determined, it was incorporated into lecture-style teaching videos, comprising three units, each lasting 12–21 minutes. Four teaching stages of TBL were employed as teaching strategies [25]. Stage 1 was pre-class preparation. Students were expected to watch teaching videos available on the teaching website at home and before class. Stage 2 was the readiness test. Students had to fill in and answer the online personal pre-class readiness test at the beginning of class. It was a structural test designed by instructors according to the curriculum objectives and contents. It covered three units and comprised 17 single-answer questions, each with four options. After the individual readiness test, a group test was conducted with the same test questions. Students in the same group used the immediate feedback assessment technique to discuss the answers by item. If an incorrect option was selected, they should continue the discussion until the correct choice was made. Stage 3 was teacher feedback and clarification. Teachers should encourage questions from students and explain course contents associated with lower correct answer rates. Stage 4 was the course application. Class activities were conducted, and homework was assigned (Table 1). Homework presentations and discussions were conducted at the end of the term.

### Instruments

#### Familiarity with attitudes toward health literacy, and confidence and implementation in communication questionnaire

The questionnaire was drawn from Chang et al.’s instruments used for a health literacy survey on community health care providers [31] with necessary revisions for differences in the participants. It included the following. First, familiarity with health literacy (9 items) covered mainly the definition of health literacy, measurement instruments, identification of those with low health literacy, the correlation between low health literacy and health outcomes, oral and written communication, and awareness of health literacy practices. Second, attitude (5 items) primarily addressed students’ perspectives on information quality in communication and universal precautions for health literacy and their views on the required competencies of medical staff for providing health literacy services and the need for health education and training. Third, confidence in oral communication (2 items) mainly tested the confidence in the correct application of teach-back and using appropriate written education materials to assist communication. Fourth, ability in written communication (4 items) mainly measured the proficiency in recognizing indicators for health literacy appropriate health educational materials and selecting, evaluating, and developing health educational materials. The questionnaire also had a scale for implementation on oral communication addressing health literacy (11 items), adopted from the teach-back items developed by the Institute for Healthcare Improvement [32]. All items were measured by self-evaluation, with points ranging from 1 to 10, where higher scores indicated better awareness, attitude, self-confidence, and implementation.

#### Curriculum satisfaction questionnaire

We measured students’ satisfaction with learning methods, the usefulness of the course content, and teaching videos. The nine-item questionnaire was self-evaluated using a scale ranging from 1 (*very dissatisfied*) to 10 (*very satisfied*). A higher score indicated higher satisfaction.

#### Reliability and validity

For this study, five experts in related fields were invited to examine the validity in terms of the clarity of questionnaire statements and the appropriateness of the tested concepts. The content validity indexes of this questionnaire ranged from 0.81 to 1.0. Cronbach’s alpha values, indicating the internal consistency of the subscales, ranged from 0.92 to 0.95.

#### Focus group interviews outline

We developed and used six semi-structured interview questions covering TBL, classroom activities, homework, health literacy topics, and internship practices to facilitate the focus group discussion.

### Research and data collection

The online questionnaire was designed to collect data. The purposes and process of the research were explained to the students before the beginning of the course. Links to the questionnaire website and consent to participate were sent by email. Students could freely decide whether to fill out the questionnaire. Next, the intervention course was offered. One week after the end of the course, students were requested to fill out the same questionnaire used for the pre-test and the course satisfaction questionnaire online. All students had a nursing practice curriculum in the hospital after completing the intervention course. One week after a three-week internship, students participated in a survey on how they adopted communication skills during the internship. Meanwhile, students who participated in the research were recruited for focus group interviews. The study invited an expert in hosting focus groups to facilitate the interviews. Asking questions according to an interview outline, the expert encouraged students to talk freely about their experience participating in the course. Each session lasted approximately 60 minutes. With prior consent from the interviewees, the interviews were recorded to generate audio data for analysis.

### Methodologies for data analysis

Quantitative data were imported into IBM SPSS Statistics 19 for descriptive statistics, presenting the degree and distribution of participants in various research variables by frequency, percentage, mean, and standard deviation. Paired *t*-tests were performed to compare the variables before and after the intervention.

Qualitative data were verbatim transcripts of interview recordings for content analysis. A data analyst first identified the behavioral units related to the research topic and then extracted, summarized, and coded them to generate concepts and categories to form an analytical framework. Another analyst randomly selected a data chunk to analyze according to the analytical framework. A comparison of the two raters’ analysis results suggested a consistency level of 0.88. For the inconsistent parts, further discussion was held until a consensus was reached.

## Results

### Demographic description of the participants

Of the 81 students who completed the pre- and post-test questionnaires, 91.4% (74) were female, and 97.5% were aged 21–25 years. Almost all of them (98.8%) had clinical learning experiences.

### Effectiveness analysis after the health literacy course

Table 2 presents a comparison of students’ outcome variables before and after the course. All four variables showed improved scores after the course.

**Table 2.** Scores before and after the course intervention.

Items	Pre-test *n* = 81	Post-test *n* = 81	*t*	*p*
	Mean ± standard deviation	Mean ± standard deviation		
Familiarity toward health literacy	5.64 ± 1.66	7.75 ± 1.34	9.12	<.001
Attitudes toward health literacy	7.43 ± 1.73	8.37 ± 1.16	4.89	<.001
Confidence in health literacy oral communication	7.36 ± 1.64	8.19 ± 1.18	4.12	<.001
written communication	5.93 ± 1.54	7.81 ± 1.38	8.83	<.001

### Analysis of the application of oral communication skills

Of the 81 participants who completed the pre- and post-test questionnaires, 67 responded to the oral communication skill survey after the internship. The results are shown in Table 3.

**Table 3.** Analysis of the application of oral communication skills.

Items	*n* = 67 Mean ± standard deviation
Use a caring tone and attitude	8.54 ± 1.33
Show a comfortable posture (look at each other and sit down while talking as much as possible)	8.36 ± 1.36
Use easy-to-understand language	8.50 ± 1.27
Ask people to repeat the message they have been told in their own words	8.36 ± 1.37
Use questions that are not embarrassing	7.89 ± 2.11
Avoid asking only yes-or-no questions	8.10 ± 1.86
Explain your responsibility to confirm the clear expression of messages	8.30 ± 1.45
Explain again if the teach-back is unsuccessful	8.47 ± 1.36
Use health literacy-friendly education materials to explain a message	8.09 ± 1.50
Record the teach-back process	7.96 ± 1.56
Include family members or caregivers in health education as necessary	8.59 ± 1.38
Overall average	8.20 ± 1.22

### Satisfaction with the health literacy course

The results for course satisfaction are shown in Table 4. Except for the reduction of the curriculum load, the average satisfaction of the other items exceeded 7.5 points. Among them, students had the highest level of satisfaction with course usefulness.

**Table 4.** Course satisfaction analysis.

Items	*n* = 81 Mean ± standard deviation
Flexible learning time and locations	7.98 ± 1.32
Possible to learn repeatedly	8.07 ± 1.22
Reduced learning burden	7.36 ± 2.05
Appropriate course length	7.77 ± 1.36
The course is extremely useful for me	8.32 ± 1.26
The teaching videos are clear and vivid	7.81 ± 1.61
The course contents are interesting	7.88 ± 1.49
The education materials are well-organized and prepared	7.94 ± 1.43
The education materials are suitably challenging	7.79 ± 1.60
Overall average	7.90 ± 1.27

### Benefits derived from the health literacy course

Data analysis for the two focus group interviews generated two categories: students’ learning of the health literacy course and their experience and feelings about TBL. Seven themes were derived from the two categories. The original statements related to the categories and themes are shown in Table 5.

**Table 5.** Qualitative analysis and summary of learning experiences with the health literacy course.

Categories	Topics	Exemplary responses
1. Learning experiences from the course and ideas on health literacy	1–1 Reflection on the problems in physician-patient communication in previous internship experiences	Example 1: ‘Most of the time, we talk quickly (for patient health education) because we are busy and regard it as a one-time task without paying attention to whether the patient really understands.’ Example 2: ‘In the nursing station where I used to practice, the health education leaflet was full of words and complicated in the description. How could a grandmother with below-elementary school education understand it?’ Example 3: ‘In the past, I may explain to the patient’s family according to my own understanding, without empathy about their situation.’
	1–2 Renewed understanding of medical communication and health education from the perspective of health literacy	Example 1: “After this course, I was aware of how to make appropriate health education leaflets according to patients’ current problems to achieve friendly communication with patients.” Example 2: ‘After class, I learned to explain simply in plain language so that people without a medical background could understand, instead of using medical terms as before.’
	1–3 Agreeable for health education materials design indicators	Example 1: ‘I think the requirements of “using everyday language” and “explaining medical jargon” are necessary. We are used to speaking in more professional terms, which cannot be understood by others and may cause misunderstanding.’ Example 2: “We did not pay attention to the words and colors of education materials. Now, we know that they are also important and may affect readers’ desire to continue reading. This indicator reminds us that these are considerably important factors.” Example 3: ‘How can people understand the key points on a leaflet at a glance? Some details, such as color distribution and word size, are relevant for a leaflet or pamphlet. [The course] was really helpful.’
	1–4 Implementation of health-literate practice during internship	Example 1: ‘(Internship in thoracic internal medicine ward) For many patients with asthma and COPD (chronic obstructive pulmonary disease), it is necessary to teach how to inhale some drugs. Some patients directly say, “yes, I know.” At this time, it is extremely important to apply the teach-back technique to ensure that they really know how to inhale before they can go home.’ Example 2: ‘Patients will go home directly after the operation here (outpatient operation room). Before they leave, the doctor will only say to them, “Do not eat hot foods, and you should eat so and so.” I think the patients are in pain and nervous after the operation, and they may not listen to what you tell them. At this time, giving them a printed health education leaflet to help explain the requirements and writing down the key points clearly would be helpful. When they reach home, with the leaflet, they could recall what the physician or nurse had told them.’
2. experiences of TBL	2–1 Preview and test before class offer the opportunity for self-study and evaluation but sometimes increase the burden	Example 1: ‘By watching the videos first, you could absorb the knowledge by yourself. The test gives you a sense of your knowledge base.’ Example 2: ‘The videos can be viewed repeatedly. In class, the teacher will explain the part that I really do not understand, and I will also pay special attention to these parts.’ Example 3: ‘You have to spend time reading in advance, but sometimes you have to deal with other courses, so you will have time pressure.’ Example 4: ‘Sometimes, there are many classes. I looked at them casually the night before and fell asleep before I finished reading them. If I did not do well in the test, I would be stressed.’
	2–2 Classroom activities provide opportunities to practice and enhance understanding	Example 1: ‘The exercise of evaluating health education materials helped us do our homework later. After practice, we learned to incorporate the indicators while doing homework (designing health education leaflets).’ Example 2: ‘Watching the videos made me aware of the physician-related problems. Discussion with classmates in relation to what we have learned in the classroom revealed that many principles for communicating with patients were not followed in the videos.’
	2–3 Gaining a sense of patient-centered communication from script design activities	Example 1: ‘(Communication script assignment) At the beginning, we would think about the dialog from the perspective of nursing students. In subsequent revisions, we incorporated the positions, mindsets, and environments of patients or their families. Thus, we understood some difficulties (in communicating) that may be encountered when people interact with professionals.’ Example 2: ‘We applied the principles of health literacy communication in the dialogs, such as using the teach-back technique. We gained a sense of health literacy communication.’ Example 3: ‘In designing the script, I thought about how to talk to patients during health education so that patients can understand.’

## Discussion

For this study, an eight-hour refined health literacy course was designed for undergraduate nursing students. TBL was accompanied by various teaching methods, including didactic, reality video simulation learning, health educational materials assessing exercise, and homework. The results showed that this approach could improve nursing students’ awareness of health literacy, attitude, and confidence in communication as expected. Students’ self-evaluation indicated that they scored up to 8 points in adopting oral communication skills during the internship.

### Course content

The course developed in this study comprised three units: introduction to health literacy, oral communication, and written communication. Further, the course covered five health literacy topics from Coleman and Appy [14]. Among the 32 priority items identified by Coleman et al. for healthcare professionals in health literacy and clear communication practices [20], at least half of the items were covered by the course. In our study, oral communication skills were included in Unit 2 of the course, with emphasis on the practice of the teach-back technique. Teach-back is an effective method for helping patients understand self-care and disease self-management [33]. While nurses often need to apply teach-back in health education, studies have indicated that the teach-back technique is often used improperly, leading to ineffective health education for patients. Nurses need more practice to apply the technique correctly [34]. Regarding the teach-back exercises designed in this study, the survey conducted after the students’ clinical internship showed that the implementation of each step of teach-back had an average of 8.20 ± 1.22 points (out of 10). The focus group interviews revealed that students can avoid using medical jargon while communicating with patients, use simple and easy-to-understand language, apply teach-back to confirm patients’ understanding, and use written health education materials to assist health education. In summary, students could apply what they learned to clinical practice.

Unit 3 of the course introduced the methods and tools for the development and evaluation of written health education materials. Notably, this unit is rarely included in the undergraduate health literacy curriculum and does not relate to items recommended by Hernes and Ott [16,21]. Nonetheless, written health education materials are often used as aids by nurses to educate patients. Studies have shown that many written health education materials are awkward in language, complex in content, and poor in readability and suitability, leading to poor comprehension among patients, who, in turn, cannot act upon the prescribed treatment plan [19,35]. Parnell proposed that developing suitable written education materials for specific audiences requires unique skills [19]. These skills have been incorporated into many evaluation tools and guidelines of health education materials for health literacy, enabling professionals to develop and evaluate written education materials and information for improving the quality of written communication [36,37]. In 2017, Taiwan’s Health Promotion Administration also published evaluation indicators and guidelines for health education materials [38] to inform the development of health literacy education materials or information.

The course incorporated relevant content, classroom activities, and homework to improve students’ written communication skills. The pre- and post-test results of the study showed that written communication ability was enhanced by the course (*t* = 8.83, *p* < .001). The results of the focus group interviews indicated that the students found the indicators of written education materials agreeable and could identify the problems with existing materials. Moreover, the students reported a positive experience in training related to written communication.

### Course hours and teaching strategies

Numerous subjects need to be covered in a health literacy course, but the time available for the course tends to be limited because of the existing overloaded undergraduate curriculum. This fact may explain why health literacy education is not widely offered [17]. In our study, the design of the health literacy course and the selection of teaching methods addressed the challenge of achieving learning objectives in a limited number of hours. The course required four hours of classroom activities, primarily TBL supplemented by other learning methods. The additional four hours were allocated to the completion of homework reports meant to enhance learning outcomes.

TBL is a way of flipped teaching [25]. By self-studying knowledge before class, students can have more time to apply the knowledge and enhance critical thinking in classroom activities [26,27]. The teaching videos offered by our study allowed students to complete the learning of knowledge by themselves before class. The health literacy competencies of identifying those with limited health literacy and applying communication skills [12] cannot be developed through didactic teaching [30]. As such, TBL, which can incorporate exercises for students to hone their communication skills, is an ideal strategy for achieving the higher-level goals in Bloom’s classification of teaching objectives, such as evaluation and application. Coleman summarized the strategies for health literacy teaching and noted that experiential learning strategies, such as workshops, small group exercises, role plays, video reviews, and standardized patients, enable learners to participate, interact, practice, and operate and are often applied to acquire skills [39]. Our study incorporated these methods into the TBL classroom activities.

For the first classroom activity, a video depicting real-world medical communication situations was reviewed. The students were reminded about the elements of health literacy communication. The video could be played repeatedly, and the instructor and students could immediately discuss communication skills. In the second classroom activity, students were divided into small groups to evaluate real-world health education leaflets against the indicators for health literacy education materials and thereby obtain a sense of skills for designing educational materials. The course included homework to offer more opportunities for honing skills. For the homework, the students were required to change roles and identify problems in physician-patient communication in a role-playing setup. They were also instructed to apply communication skills to the script design. The change in roles was expected to allow each participant to practice important communication skills and learn from mistakes and thus know how to communicate properly [39]. The results showed that the students regarded the course as practical because they could identify the problems caused by limited health literacy in medical communication. The course also renewed their understanding of skills for medical communication and health education from the perspective of health literacy. They were impressed by the classroom activities and assignments and realized the importance of patient-centered communication. Both quantitative and qualitative data suggested that the health literacy course had positive effects on improving students’ skills and attitudes.

### Pre-class preparation

With teaching videos available before class, students could learn at their own pace. For complex concepts, they could fast forward, pause, or repeat the video. Autonomy in learning time also made teaching more flexible [40]. In our study, the teaching videos were recorded and made available online. The survey on learning satisfaction indicated that overall course satisfaction was 7.90 ± 1.27. Students were satisfied with the course videos and online learning approach. They appreciated the flexible learning time and locations in preparing classroom activities by using online teaching videos, repeatable learning, and appropriate course content. The analysis of qualitative data also echoed the findings in the literature: online preview makes it possible to watch the videos repeatedly and learn at one’s own pace. However, some students also mentioned that they needed to arrange time to preview before class and regarded it as a burden, given their limited free time. In our study, three teaching videos were produced, with lengths of 12, 14, and 21 minutes – exceeding the standard of 8–12 minutes suggested by Sams and Bennett [41]. The videos were deemed a heavy learning burden, probably because of their length. In the future, simplifying the content and shortening the length of videos may reduce the time pressure of preview sessions.

## Limitations

This study has several limitations. First, we aimed to test the effectiveness of an intervention course. However, without a control group, we could not state confidently that the change in outcomes over time is because of the intervention measures. Second, the self-rating scales used may have led to biased results because the respondents may have answered the questions faithfully but under the effects of social expectations [42]. Third, the course evaluation focused on the performance of nursing students. Indicators such as the improvement of clients’ health literacy and reduction of communication barriers were not considered. Future research can incorporate indicators related to clients in evaluating course effectiveness. Moreover, tracking the application of health literacy communication skills after employment may provide further insights into the long-term benefits of the course. Finally, there may be some hurdles to the promotion of the course. In our study, the instructor has many years of experience in health literacy research and teaching. Nevertheless, as Scott noted, many nursing instructors are not familiar with the topic of health literacy and are not aware of its importance, thereby needing training [22]. Existing problems with nursing education in Taiwan may also make it challenging to promote the course. Therefore, the training or in-service training of nursing instructors on health literacy should be considered.

## Conclusion

There is no consensus on health literacy competencies and education for undergraduate nursing students. More research is needed to inform such an agreement. In this study, we designed a refined health literacy course with structural curriculum contents and teaching method recommendations. The scope of the contents was consistent with that suggested in the literature for undergraduate nursing students. TBL and other teaching techniques were adopted, considering the limited course hours and variety of knowledge and skills to be acquired. The effectiveness evaluation suggested that the course performed as expected. Nursing education institutions that cannot provide health literacy education owing to excessive curriculum burden may find this course especially valuable.
